# Cross‐cultural adaptation of the FRAIL scale for critically ill patients in Spain

**DOI:** 10.1002/nop2.2011

**Published:** 2023-09-29

**Authors:** Susana Arias‐Rivera, María Nieves Moro‐Tejedor, Marta Raurell‐Torredà, Irene Cortés‐Puch, Fernando Frutos‐Vivar, Cristina Andreu‐Vázquez, María Mar Sánchez‐Sánchez, Raquel Sánchez‐Izquierdo, Lorena Oteiza‐López, Sonia López‐Cuenca, Marta Checa‐López, Raquel Jareño‐Collado, Virginia López‐López, Eva Isabel Sánchez‐Muñoz, Luis Fernando Carrasco Rodríguez‐Rey, María Jesús Frade‐Mera, Rebeca Padilla‐Peinado, Alejandro Huete‐García, Amanda Lesmes‐González Aledo, Federico Gordo‐Vidal, Ana Rodríguez‐Merino, Mónica Vázquez‐Calatayud, Gloria Vázquez‐Grande, Dolores Mateo, Raquel Herrero‐Hernández

**Affiliations:** ^1^ Department of Nursing Research Hospital Universitario de Getafe Madrid Spain; ^2^ Nursing Research Support Unit, General University Hospital, Gregorio Marañón Gregorio Maranon Health Research Institute (IiSGM) Madrid Spain; ^3^ Red Cross University School of Nursing Autonomous University of Madrid Madrid Spain; ^4^ Faculty of Medicine and Health Sciences Universidad de Barcelona Barcelona Spain; ^5^ Division of Pulmonary, Critical Care and Sleep Medicine University of California Davis Medical Center (EEUU) Sacramento California USA; ^6^ Intensive Care Unit Hospital Universitario de Getafe Madrid Spain; ^7^ Department of Medicine, Faculty of Biomedical and Health Sciences Universidad Europea de Madrid Madrid Spain; ^8^ Geriatric Department Hospital Universitario de Getafe Madrid Spain; ^9^ Critical Cardiology Care Unit Hospital Universitario 12 de Octubre Madrid Spain; ^10^ Department of Nursing, Faculty of Nursing, Physiotherapy, and Podology Complutense University of Madrid Madrid Spain; ^11^ Intensive Care Unit Hospital Universitario 12 de Octubre Madrid Spain; ^12^ Intensive Care Unit Hospital Virgen de la Salud Toledo Spain; ^13^ Intensive Care Unit HM Hospitales Madrid Spain; ^14^ Intensive Care Unit Hospital Universitario del Henares Madrid Spain; ^15^ Grupo estable de investigación en Patología Crítica. Facultad de Medicina Universidad Francisco de Vitoria Madrid Spain; ^16^ Royal Brompton and Harefield Hospital Trust London UK; ^17^ Area of Nursing Professional Development Clínica Universidad de Navarra Pamplona Spain; ^18^ Faculty of Nursing University of Navarra Pamplona Spain; ^19^ Navarra Institute for Health Research (IdiSNA) Pamplona Spain; ^20^ Section of Critical Care Medicine, Department of Medicine University of Manitoba Winnipeg Manitoba Canada; ^21^ Intensive Care Unit, Broomfield Hospital Mid Essex NHS Foundation Trust Chelmsford UK

**Keywords:** adults, critical care, cross‐cultural comparison, frailty, nurse, nursing, translating

## Abstract

**Aim:**

To translate and culturally adapt the FRAIL scale into Spanish and perform a preliminary test of diagnostic accuracy in patients admitted to intensive care units.

**Design:**

Cross‐sectional diagnostic study.

**Methods:**

Five intensive care units (ICU) in Spain were participated. Stage 1: Three native Spanish‐speaking bilingual translators familiar with the field of critical care translated the scale from English into Spanish. Stage 2: Three native English‐speaking bilingual translators familiar with critical care medicine. Stage 3: Authors of the original scale compared the English original and back‐translated versions of the scale. Stage 4: Five nurses with more than 5 years of ICU experience and five critical care physicians assessed the comprehension and relevance of each of the items of the Spanish version in 30 patients of 3 different age ranges (<50, 50–65 and >65 years).

**Results:**

The FRAIL scale was translated and adapted cross‐culturally for patients admitted to intensive care units in Spain. The process consisted of four stages: translation, back translation, comparison and pilot test. There was good correspondence between the original scale and the Spanish version in 100% of the items. The participating patients assessed the relevance (content validity) and comprehensibility (face validity) of each of the items of the first Spanish version. The relevance of some of the items scored low when the scale was used in patients younger than 65 years.

**Conclusions:**

We have cross‐culturally adapted the FRAIL scale, originally in English, to Spanish for its use in the critical care medical setting in Spanish‐speaking countries.

**Implications for Professionals:**

Physicians and nurses can apply the new scale to all patients admitted to the intensive care units. Nursing care can be adapted according to frailty, trying to reduce the side effects of admission to these units for the most fragile patients.

**Reporting Method:**

The manuscript's authors have adhered to the EQUATOR guidelines, using the COSMIN reporting guideline for studies on the measurement properties of patient‐reported outcome measures.

**Patient or Public Contribution:**

In a pilot clinical study, we applied the first version of the FRAIL‐Spain scale to intensive care unit (ICU) patients. Five nurses with more than 5 years of ICU experience and five critical care physicians assessed the relevance (content validity) and comprehensibility (face validity) of the five items of the first Spanish version. Relevance was assessed using a 4‐point Likert scale ranging from 1 (no relevance) to 4 (high relevance), and comprehensibility was assessed as poor, acceptable or good. Each health professional applied the scale to three patients (total number of patients = 30) of three different age ranges (<50, 50–65 and >65 years) and recorded the time of application of the scale to each patient. Although the frailty scales were initially created by geriatricians to be applied to the elders, there is little experience with their application in critically ill patients of any age. Therefore, more information is needed to determine the relevance of using this scale in critical care patients. In this pilot study, we considered that nurses and critical care physicians should evaluate frailty using this adapted scale in adult patients admitted to the Intensive Care Units.

## 1 INTRODUCTION

Frailty represents the clinical expressions of biological changes and cumulative deficits that occur with aging (Athari et al., 2019; De Biasio et al., 2020). Despite a lack of consensus on the clinical definition of frailty, it is considered a syndrome that includes an aging‐associated decline in reserve and function of multiple physiological systems and the appearance of chronic or acute diseases in older adults (Abellan van Kan, Rolland, Bergman, et al., 2008; Abellan van Kan, Rolland, Morley, & Vellas, 2008; Athari et al., 2019; Dent et al., 2019). The older population is associated with worse quality of life (Crocker et al., 2019) and a higher risk of adverse outcomes in response to stressors (Abellan van Kan, Rolland, Morley, & Vellas, 2008). Frailty has also been applied to other age groups because functional decline can also occur as a complication of medical conditions or diseases (Morley et al., 2013),

With the increased life expectancy and technological advances, the number of patients with comorbidities admitted to intensive care units (ICUs) has increased. Indeed, frailty is present in up to 30% of patients admitted to the ICU (Muscedere et al., 2017) and is associated with prolonged length of stay, increased risk of readmission and long‐term mortality (Bagshaw et al., 2016; Hill et al., 2021; López Cuenca et al., 2019; Muscedere et al., 2017). Most ICU prognostic scores assess the severity of illness and predict short‐term mortality based on the patient evolution over the first 24 h in ICU, Acute Physiology and Chronic Health Evaluation, Sequential Organ Failure Assessment or Simplified Acute Physiology Score (McDermid et al., 2011). Nevertheless, there is an increasing interest in assessing other outcomes relevant for patients and their families, such as short‐ and long‐term intensive care‐related mortality and resulting quality of life (Desai & Gross, 2019; Gordo et al., 2018). To correctly estimate those outcomes, we must consider the patient's functional status or frailty before admission.

The fact that there are up to 67 heterogeneous frailty assessment scales (Buta et al., 2016) makes it challenging to know the prevalence of frailty and its impact on society (Theou et al., 2013). Most frailty scales—Fried frailty phenotype, Edmonton Frailty Scale, Tilburg Frailty Indicator, Gerontopole Frailty Screening Tool, PRISMA‐7, Groningen Frailty Indicator, individual frailty measures; Sherbrooke Postcard Questionnaire, Clinical Frailty Scale and FRAIL scale—are based on the assessment of motor function, gait, independence and muscle strength. Due to the severity and heterogeneity of patients admitted to the ICU, applying the actual scales is complicated. Usually, interaction with critically ill patients is complex or impossible, so the frailty degree should be assessed using simple scales, whose information could be retrieved from the clinical history or with simple questions to relatives or close friends. In this line, the most commonly used scales in the intensive care setting are the Clinical Frailty Scale (Rockwood et al., 2005) and the FRAIL scale.

## 2 BACKGROUND

In 2007, a panel of experts from Europe, USA and Canada, in frailty developed the FRAIL scale (Abellan van Kan, Rolland, Bergman, et al., 2008; Abellan van Kan, Rolland, Morley, & Vellas, 2008) that assesses frailty by evaluating five domains: Fatigue, Resistance, Ambulation, Illnesses/comorbidities and Loss of weight. The name FRAIL derives from the first letter of each of the items. Since published, the FRAIL scale has been translated into several languages (German (Braun et al., 2018), Chinese (Dong et al., 2018), Korean (Jung et al., 2016) and Mexican Spanish (Rosas‐Carrasco et al., 2016)), adapted to different sexes, age ranges and cultures and applied using different methods (by telephone or by email) validated in many studies (Dong et al., 2018; Gardiner et al., 2015; Jung et al., 2016; Kojima, 2018a, 2018b; Li et al., 2015; Morley et al., 2012; Papachristou et al., 2017; Ravindrarajah et al., 2013; Rosas‐Carrasco et al., 2016; Susanto et al., 2018; Woo et al., 2012).

Adapting the FRAIL scale to ICU patients would let us know their frailty status at ICU admission, helping to make better clinical decisions and allocate resources that potentially improve patient outcomes. Since communication with ICU patients is not always possible, patient information was obtained from their medical records and/or family members.

Therefore, appropriate instruments and tools are needed to maintain and improve the quality of healthcare and research. This study aimed to translate and culturally adapt the FRAIL scale into Spanish society and adapt it to critically ill patients admitted to ICUs in Spain.

## 3 METHODS

### 3.1 Participants and data collection

The FRAIL scale was translated and cross‐culturally adapted for its use in patients admitted to ICUs in Spain, while preserving similar semantic, conceptual and technical equivalencies of the evaluation criteria to the original one. The process involved four stages: translation, back translation, comparison and pilot testing (Sousa & Rojjanasrirat, 2011). Although the FRAIL scale does not fall under copyright protection, we obtained authorization from the authors of the original scale, who were also involved in comparing the original and the newly adapted scale.

The FRAIL scale has five domains that form its acronym: Fatigue, Resistance (defined as the ability to climb stairs), ambulation (ability to walk a certain distance), Illnesses and Loss of weight (>5%; Table 1). The scale score ranges from 0 to 5 points based on the presence or absence of each item. Depending on the final score, the patients are stratified into three categories: good health and absence of frailty (0 points), pre‐frailty (1–2 points) and frailty (3–5 points) (Abellan van Kan, Rolland, Morley, & Vellas, 2008).

**TABLE 1:** Adaptation of the FRAIL scale into FRAIL‐Spain.

Original items	Original definition	Spanish definition	Spanish items
Fatigue	‘How much of the time during the past 4 weeks did you feel tired?’ 1 = All of the time, 2 = Most of the time, 3 = Some of the time, 4 = A little of the time, 5 = None of the time. Responses of ‘1’ or ‘2’ are scored as 1 and all others as 0.	¿Cuánto tiempo se sintió cansado durante las últimas 4 semanas? 1 = todo el tiempo o la mayor parte del tiempo, 0 = algunas veces, pocas veces o nunca.	Fatiga
Resistance	‘By yourself and not using aids, do you have any difficulty walking up 10 steps without resting?’ 1 = Yes, 0 = No.	¿Tiene alguna dificultad para subir, solo y sin ayuda, 10 escalones sin descansar? (no se considera ayuda el uso habitual del bastón) 1 = Sí, 0 = No.	Resistencia
Ambulation	‘By yourself and not using aids, do you have any difficulty walking several hundred yards?’ 1 = Yes, 0 = No.	¿Tiene alguna dificultad para caminar, solo y sin ayuda, varios cientos de metros? 1 = Sí, 0 = No.	Ambulación
Illnesses	For 11 illnesses, participants are asked, ‘Did a doctor ever tell you that you have [illness]?’ 1 = Yes, 0 = No. The total illnesses (0–11) are recoded as 0–4 = 0 and 5–11 = 1. The illnesses include hypertension, diabetes, cancer (other than a minor skin cancer), chronic lung disease, heart attack, congestive heart failure, angina, asthma, arthritis, stroke and kidney disease.	Se les pregunta a los participantes a cerca de 11 enfermedades: ¿Alguna vez un médico le ha dicho que tiene [enfermedad]?, las enfermedades incluidas son: hipertensión, diabetes, cáncer (que no sea un cáncer de piel menor), enfermedad pulmonar crónica, infarto de miocardio, insuficiencia cardíaca congestiva, angina, asma, artritis, ictus y enfermedad renal. 1 = 5 o más enfermedades, 0 = menos de 5 enfermedades.	Enfermedades
Loss of weight	‘How much do you weigh with your clothes on but without shoes? [current weight]’ ‘One year ago in (MO, YR), how much did you weigh without your shoes and with your clothes on? [weight 1 year ago]’ Percent weight change is computed as: [[weight 1 year ago – current weight]/[weight 1 year ago]] * 100. Percent change >5 (representing a 5% loss of weight) is scored as 1 and <5 as 0.	¿Ha perdido usted peso involuntariamente en el último año (≥5%) o ha notado que la ropa le queda más holgada? 1 = Sí, 0 = No.	Pérdida de peso no intencionada

*Note*: Final score out of FRAIL‐Spain. Patients are stratified into three categories: good health and absence of frailty (0 points), pre‐frailty (1–2 points) and frailty (3–5 points).

#### 3.1.1 Stage 1: Translation of the original FRAIL scale and generation of the first version of the FRAIL‐Spain scale

Three bilingual translators, speaking Spanish as a native language and familiar with critical care medicine, translated the scale from English into Spanish, working independently. Each translator was provided with a brief description of the scale's characteristics and uses and the main goal of the scale translation. After evaluating their semantic equivalences, an expert committee harmonized the three resulting translations into the first Spanish version of the FRAIL scale (FRAIL‐Spain‐v1). This expert committee consisted of five bilingual healthcare workers: two intensive care nurses, two intensive care physicians and one geriatrician with expertise in frailty.

#### 3.1.2 Stage 2: Back translation from de FRAIL‐Spain scale to an English FRAIL scale

Three bilingual translators, speaking English as a native language and familiar with the field of critical care, performed a backward translation of the second Spanish version of the scale (FRAIL‐Spain‐v2 from stage 2) into English. The three translators worked independently and were aware of the purpose of the scale but unfamiliar with its original English version. As in the previous stage, the expert committee harmonized the three back translations, generating a single English version.

#### 3.1.3 Stage 3: Comparison of the two FRAIL English scales

The authors of the original scale compared the English original and back‐translated versions of the scale. Each scale item's semantic, technical and conceptual equivalences were assessed as good, appropriate or bad, which helped to obtain the final version of the FRAIL‐Spain scale.

#### 3.1.4 Stage 4: Pilot test and development of the second version of the FRAIL‐Spain scale

In a clinical pilot study, we applied the first version of the FRAIL‐Spain scale to ICU patients. Five nurses with more than 5 years of experience in ICU and five critical care physicians assessed the relevance (content validity) and comprehensibility (face validity) of the five items in the first Spanish version. The relevance was evaluated using a 4‐point Likert scale ranging from 1 (no relevance) to 4 (high relevance), and the comprehensibility was considered bad, acceptable or good. Each health practitioner applied the scale to three patients (total number of patients = 30) of three different age ranges (<50, 50–65 and >65 years old) and recorded the time of the scale application to each patient. We consulted the original scale's authors to resolve any outstanding questions. After incorporating all their feedback, the expert committee developed the second Spanish version of the scale (FRAIL‐Spain‐v2).

### 3.2 Statistical analysis

Quantitative variables are shown as medians and 25–75th interquartile range (IQR) or means and standard deviation (SD), and categorical variables were reported as absolute (*n*) and relative (%) frequencies. Shapiro–Wilk test was used to assess normality. The Mann–Whitney U‐test was used to compare medians for quantitative variables and the chi‐square test was used for categorical variables. A two‐tailed *p* value of <0.05 was considered statistically significant. Statistical analyses were performed using IBM SPSS Statistics for Windows (version 26.0. IBM Corp.).

### 3.3 Ethics

The study protocol was reviewed and approved by the Ethics Committee for Drug Research (CEIm) of the Hospital (IRB number: CEIm2019/42). The requirement for informed consent from individual patients was waived because the study design did not include the registration of patients' data.

## 4 RESULTS

After stage 1, translation and stage 2, back translation, the non‐original English version was obtained and sent to the scale's authors to evaluate its concordance (stage 3). The concordance for the five items between the back‐translated versions and the original English scale was considered ¨good¨ by the latter's authors.

**FIGURE 1:**
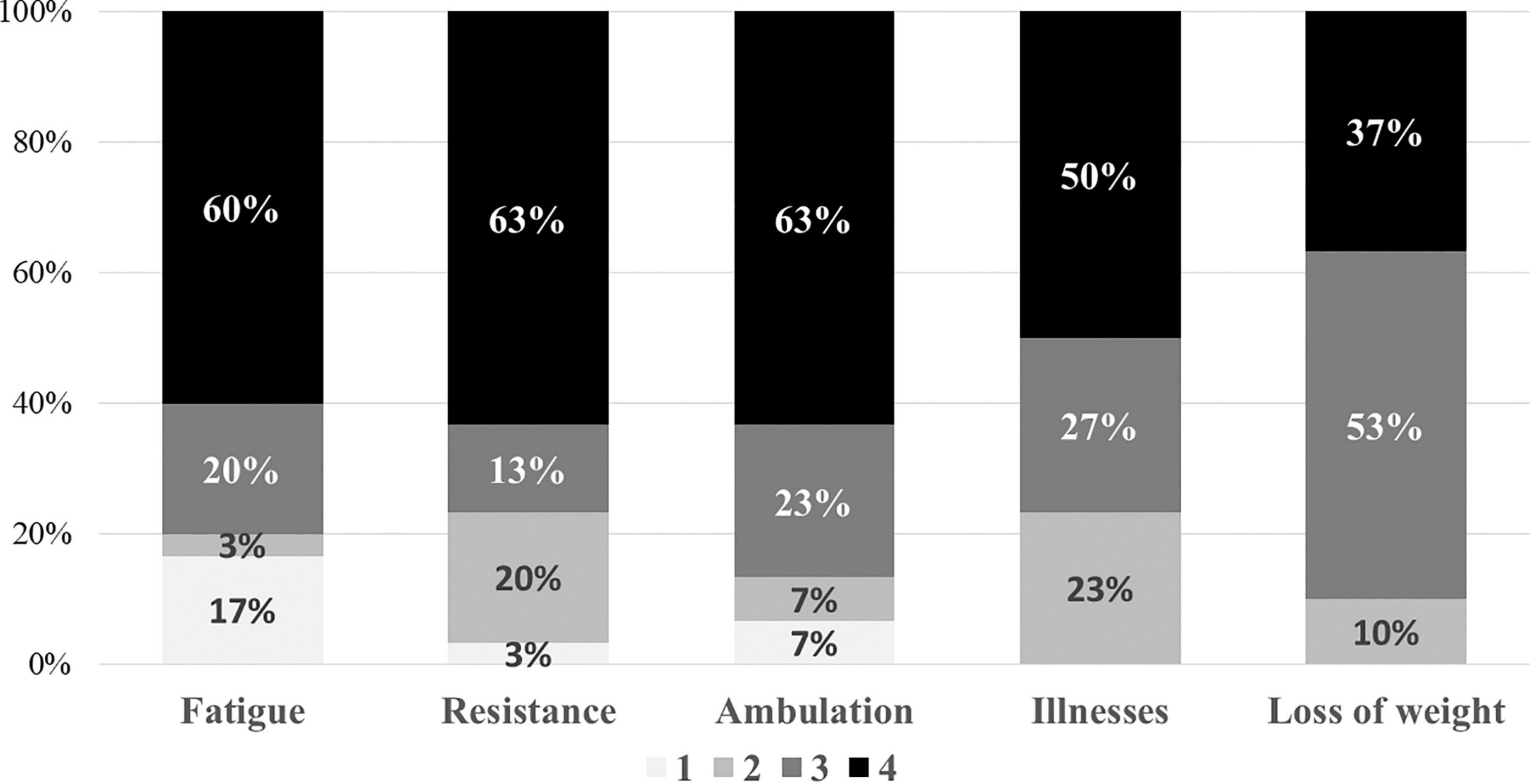
Relevance assessments for each of the items of the scale.

**FIGURE 2:**
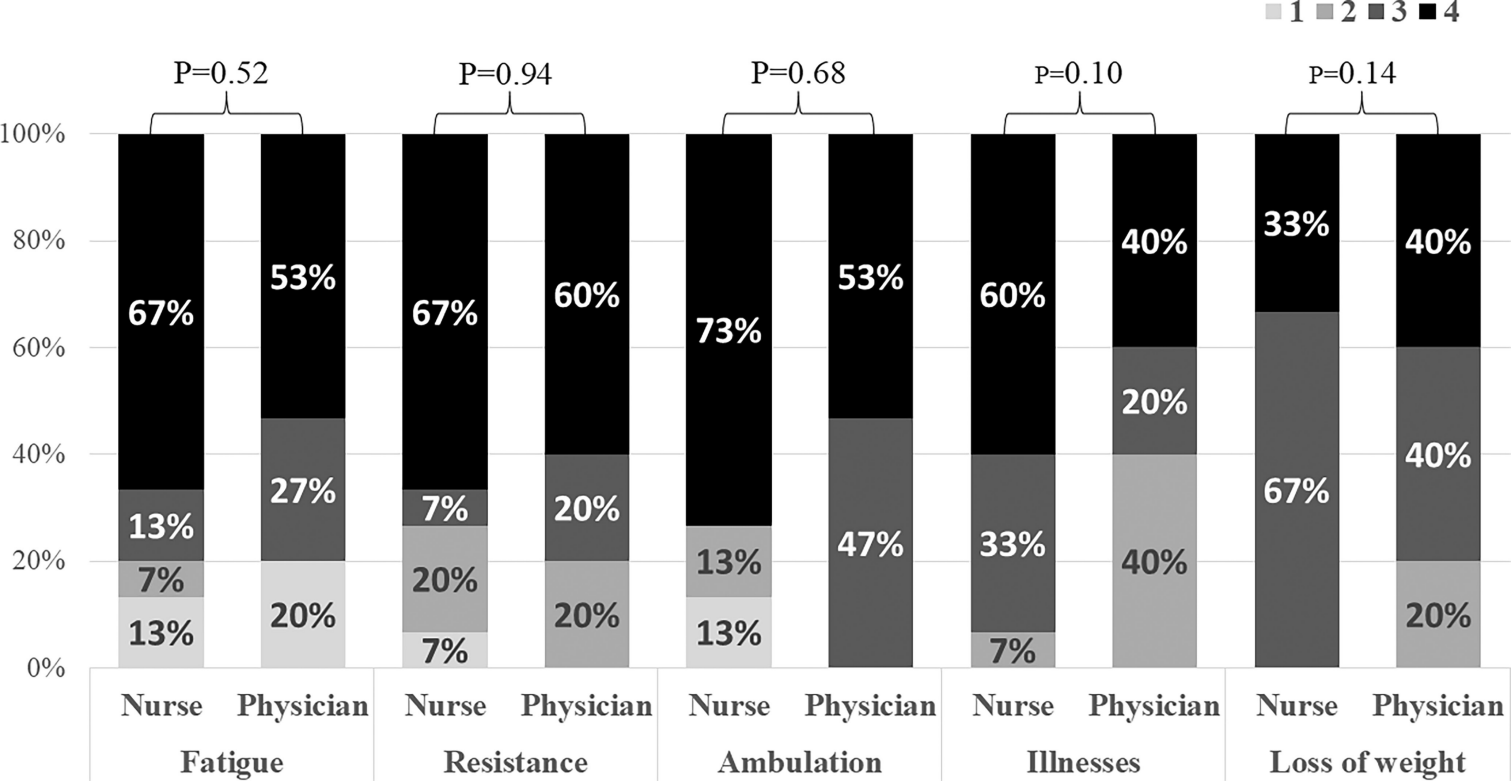
Relevance assessments for each of the items of the scale, by different type of health practitioners.

**FIGURE 3:**
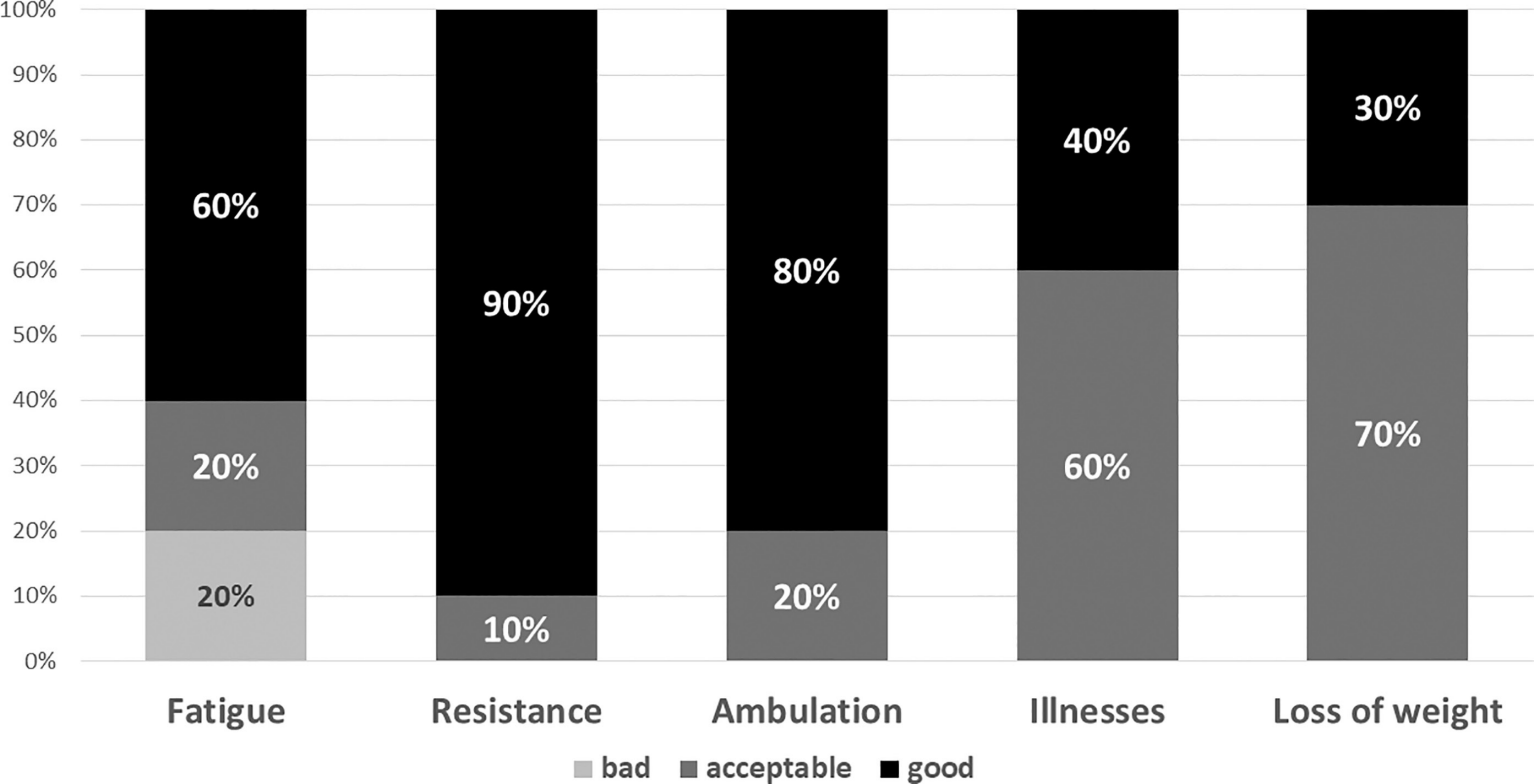
Comprehensibility assessment for each of the items of the scale.

In the clinical pilot study (stage 4), we applied the first version of the FRAIL‐Spain scale to ICU patients and obtained 30 evaluations from 10 health practitioners. The relevance of fatigue obtained the maximum score (4 points) in 60% of the evaluations and 3 points in the remaining 20% (Figure 1). The median (IQR) score was 4 points (3–4 points), with no statistically significant differences between health practitioners (nurses vs. physicians, median [IQR]: 4[3–4] vs. 4[3–4]; *p* = 0.52) (Figure 2). Comprehensibility of this item was evaluated as good by 60% of the practitioners (80% of nurses and 40% of physicians) (Figure 3). Those who did not evaluate it as good considered the item too subjective and only answerable by the patients themselves. In addition, the presence of five possible responses received a negative evaluation. Therefore, the expert committee modified the text to a binary response (Table 1).

The relevance of resistance scored 4 points in 63% of the evaluations (Figure 1) and was considered to have good comprehensibility by 90% of the health practitioners (Figure 3). The median (IQR) score for relevance was 4 points (2.8–4 points) (nurses vs. physicians, median [IQR]: 4[2–4] vs. 4[3–4], *p* = 0.94). We consulted the original scale's authors about comprehensibility‐related questions, and consequently, we slightly modified the content of this item. As a result, we accepted that the ascent of 10 stairs must be without stops and that a walking aid was allowed if the patient previously used one.

The relevance of ambulation scored 4 points in 63% of the evaluations (73% of nurses and 53% of physicians; Figures 1 and 2). The median score (IQR) was 4 points (3–4 points; nurses vs. physicians, median [IQR]: 4[2–4] vs. 4[3–4], *p* = 0.68). Comprehensibility of this item was considered good by 80% of the evaluators (Figure 3). The received comments did not result in any statistically significant modification of the translated or harmonized text; only yards were converted to meters.

Interestingly, these three items—fatigue, resistance and ambulation‐ received the lowest score of relevance when the scale was applied to patients younger than 65. Even in some cases, relevance scored lower for patients aged 50–65 years compared to patients younger than 50 years.

The relevance of the illnesses item scored 4 and 3 points in 50% and 27% of the evaluations, respectively (Figure 1). In total, 93% of nurses versus 60% of physicians scored 3 or 4 points for relevance (Figure 2). The median (IQR) score was 3.5 points (2.8–4 points) (nurses vs. physicians, median [IQR]: 4[3–4] vs. 3[2–4], *p* = 0.10). Comprehensibility was considered acceptable by 60% of the evaluators and good by 40% (Figure 3). The reviewers suggested that asking a first question about the presence or absence of illnesses with a binary answer followed by a question about the total number of illnesses could be confusing. Therefore, the expert committee modified the text unifying both questions and including only a binary response (Table 1).

The relevance of weight loss scored 3 and 4 points in 53% and 37% of the evaluations, respectively (Figure 1). The median (IQR) score was 3 points (3–4 points) (nurses vs. physicians, median [IQR]: 3[3–4] vs. 3[3–3], *p* = 0.22). Comprehensibility was considered acceptable by 65% of the health practitioners and good by the remaining 30% (Figure 3). This item caused multiple comments and questions, which were communicated to the original scale's author. The most frequent comment referred to the difficulty in evaluating weight loss when the patients (or families) did not know the patient's weight in the previous year. In addition, some questions arose about the convenience of including the weight loss caused by a voluntary diet and whether a minimum of 5% weight loss was required to score.

The median time required for the scale application was 6 min (IQR, 3–6 min) and included the patient/family interview and the review of the medical records. There were no statistically significant differences in time between nurses and physicians, with a median of 4 min (IQR, 3–7 min) versus 5 min (IQR, 3–9 min), respectively.

All the questions and comments from this pilot study were transferred to the original scale's authors, who considered modifying the text for clarity in order to facilitate the scale assessment. These modifications were evaluated as good by the health practitioners involved in the pilot test (Table 1). All these modifications resulted in a second version of the Spanish FRAIL scale (FRAIL‐Spain‐v2).

## 5 DISCUSSION

Frailty is a clinical condition associated with prolonged length of stay, increased risk of re‐admission and higher mortality rates in critically ill patients admitted to the ICU (Muscedere et al., 2017). Knowing the frailty status of the patient prior to the ICU admission is important for the clinical decision‐making and resource allocation. However, there is no frailty scale adapted adequately to the ICU patients. In the present study, we have developed a frailty scale for patients admitted to Spain's ICUs based on the original FRAIL scale (Abellan van Kan, Rolland, Bergman, et al., 2008). This derived Spanish scale—the FRAIL‐Spain scale—showed semantic, technical, conceptual and criterion equivalence to the original scale. Assessing the validity and reliability of these scales is essential for their clinical implementation. Also, adapting these scales to the clinical setting and the cultural and linguistic characteristics of the patient population is one of the first processes needed before their implementation (Beaton et al., 2000; Sousa & Rojjanasrirat, 2011).

For the FRAIL score adaptation, we followed the recommendations of Sousa and Rojjanasrirat (2011) with some slight modifications. Given that the resulting scale was intended to be used in critically ill patients, all the translators of the different versions of the scale were ICU practitioners (nurses and physicians). Non‐ICU professionals, however, were part of the expert committee that harmonized the translations.

Although we intended to keep the items as close to the original scale as possible, some changes were required because of social and cultural implications. Therefore, the editing process not only consisted of a direct translation from English into Spanish of the original version but also its adaptation to a different clinical, social and cultural setting. As reported by Dong et al. (2018) in their adaptation to the Chinese population, the response to the fatigue item of our Spanish scale was dichotomized. Among the different published versions of the FRAIL scale, the most remarkable changes we made were in the ¨weight loss¨ domain. Whereas it was unchanged by Rosas‐Carrasco et al. (2016) in their Mexican Spanish adaptation, Theou et al. took into account the decrease in appetite and food consumption instead of weight loss (Theou et al., 2013). In our adaptation, we maintained the weight loss criterion and, as an alternative, we added the perception of clothing looser.

Interestingly, the relevance of some of the criteria scored low when the scale was applied to patients younger than 65 years. The concept of frailty itself is commonly associated with the older population, and this perception could influence the results of the pilot test in our study. In this line, the health practitioners participating in the pilot test considered assessing fatigue, resistance and ambulation in younger patients less relevant than in older patients, probably because they may consider these criteria associated with aging. These opinions, however, are contrary to the results of Rosas‐Carrasco's study showing a low correlation between the scores for these items and age (Rosas‐Carrasco et al., 2016). Therefore, we considered it necessary to maintain these items in all cases, even when the scale is applied to younger patients.

The differences observed between nurses and physicians in the relevance scores of the illnesses item may occur because physicians systematically assess comorbidities on admission and focus on the diagnosis and management of the current patient disease, using validated clinical scales and scores and, consequently, the inclusion of an additional scale loses its relevance. In contrast, nurses might consider this information more relevant when assessing frailty because, while being aware of patient comorbidities, they may not incorporate this information into their daily patients' goals of care as much as physicians do for their clinical decision‐making; nurses train to be patient‐focused with a holistic lens.

The median time to apply the scale is relatively short, considering that it includes the time to review the patient's medical records. On the other hand, this time reflects, at least in part, the need for familiarity with using the scale in critically ill patients. This time may shorten as the scale becomes more frequently used. Nevertheless, the time required in our study is similar to that reported by Jung et al. (2016), being less than 3 min in 72.8% of the evaluations, 3–5 min in 23.3% and 5–10 min in the remaining evaluations.

### 5.1 Relevance to clinical practice, research and organizational field

There is controversy about using frailty scales for ICU triage (Flaatten, Beil, & Guidet, 2020; Flaatten, Van Heerden, et al., 2020). As suggested by Darvall et al. (2020), frailty scales should not be used as a prognostic tool for short‐term mortality but rather as an additional variable in the global patient assessment, particularly when ICU demand exceeds health resource availability. In this line, assessing frailty in patients admitted to the ICU can influence the clinical decisions and goals of care and help allocate resources to those patients who may benefit most. The primary and long‐term goal of all these decisions must be preserving the quality of life and allowing the patients to return to their previous clinical status following the ICU stay. We considered the adaptation of the frailty scale to the ICU environment as the initial step needed before incorporating it into the systematic assessment of critically ill patients on ICU admission.

The availability of a reliable tool to assess frailty at the ICU admission will allow us to determine whether the baseline functional assessment can influence the clinical evolution and the clinical status at ICU and hospital discharge. Also, it can help find out the factors that can influence and affect frailty during ICU and hospital stays. Finally, it could help us investigate which care we must implement to avoid developing or worsening frailty. A proper assessment of the frailty degree of the patient before ICU admission would allow us to recognize those patients at higher risk of complications and to provide earlier and specific care to improve the clinical status of the patients for their recovery. In this line, the application of this scale can be particularly relevant in those patients scheduled to be transferred to the ICU, for example, after major surgery. We must perform this evaluation in different patient cohorts according to their frailty and age.

### 5.2 Strengths and limitations

Our study can have some limitations. First, health practitioners were unfamiliar with the scale and not frailty experts, which could affect the scale assessment of relevance. It is worth noting, however, that the rigorous process used for translation and cross‐cultural adaptation of the scale (Sousa & Rojjanasrirat, 2011) yielded a valuable instrument to be used in different languages and clinical contexts while maintaining the semantic, conceptual, technical and criterion equivalence to the original scale. Second, the opinion of some of the professionals who considered fatigue, endurance and ambulation to be of little relevance in patients aged 50–65 years. These items were maintained in the new scale because other authors did not observe a correlation between these items and age (Rosas‐Carrasco et al., 2016). Nevertheless, the analysis of the validation of the scale in that age group could clarify its relevance and determine whether it is necessary to modify the scale or advise against its use in this age group.

## 6 CONCLUSIONS

We have translated and cross‐culturally adapted the FRAIL scale from English into Spanish to be used in critical care patients, in the ICUs of Spain, in different age ranges (<50, 50–65 and >65 years). Its application requires no training and can be completed in 6 min.

## AUTHOR CONTRIBUTIONS

SAR involved in conceptualization, software, formal analysis, data curation, writing the original draft, supervision, project administration and funding acquisition. SAR, MNMT and MRT involved in methodology. SAR, MNMT; MRT, ICP, FFV, CAV, MMSS, RSI, LOL, SLC, MCL, RJC, VLL, EISM, LFCRR, MJFM, RPP, AHG, ALGA, FGV, ARM, MVC, GVG, MD and RHH involved in investigation, review, editing and validation. SAR, ICP, FFV, CAV, MMSS, RSI, LOL, SLC, MCL, RJC, VLL, EISM, LFCRR, MJFM, RPP, AHG, ALGA, FGV, ARM, MVC, GVG and MD provided the resources. SAR, ICP, FFV and RHH involved in visualization. All the authors reviewed the final manuscript before submitting for publication and critically revised for important intellectual content.

## FUNDING INFORMATION

The present work was supported by the Ministry of Economy and Competitiveness ISCIII‐FIS grant PI20/01231. The authors have checked to make sure that our submission conforms as applicable to the Journal's statistical guidelines described here. There is a statistician on the author team Cristina Andreu‐Vázquez.

## CONFLICT OF INTEREST STATEMENT

The authors declare no conflict of interest.

## ETHICS STATEMENT

The study protocol was reviewed and approved by the Ethics Committee for Drug Research (CEIm) of the Hospital Universitario de Getafe (IRB number: CEIm2019/42).

## Data Availability

The data would be available on request from a researcher upon submission of a project.
