# Correction to ‘Cross‐Cultural Adaptation of the FRAIL Scale for Critically Ill Patients in Spain’

**DOI:** 10.1002/nop2.70696

**Published:** 2026-07-31

Arias‐Rivera, S. , M. N. Moro‐Tejedor , M. Raurell‐Torredà , et al. 2023. “Cross‐Cultural Adaptation of the FRAIL Scale for Critically Ill Patients in Spain.” Nursing Open, 10, no. 12: 7703–7712. 10.1002/nop2.2011.37775964 PMC10643834

In the **Funding information**, the text “The present work was supported by the Ministry of Economy and Competitiveness ISCIII‐FIS grant PI20/01231” was incorrect. This should have read: “***Funded by grant from the Spanish Ministry of Economy, Industry and Competitiveness, co‐financed by the European Regional Development Funds (Instituto de Salud Carlos III, PI20/01231)***.”

We apologize for this error.

